# Hyperchloremic Metabolic Acidosis due to Cholestyramine: A Case Report and Literature Review

**DOI:** 10.1155/2015/309791

**Published:** 2015-09-03

**Authors:** Fareed B. Kamar, Rory F. McQuillan

**Affiliations:** ^1^University of Calgary, Suite G15, 1403-29 Street NW, Calgary, AB, Canada T2N 2T9; ^2^University of Toronto and University Health Network, Toronto General Hospital, Room 8N-842, 200 Elizabeth Street, Toronto, ON, Canada M5G 2C4

## Abstract

Cholestyramine is a bile acid sequestrant that has been used in the treatment of hypercholesterolemia, pruritus due to elevated bile acid levels, and diarrhea due to bile acid malabsorption. This medication can rarely cause hyperchloremic nonanion gap metabolic acidosis, a complication featured in this report of an adult male with concomitant acute kidney injury. This case emphasizes the caution that must be taken in prescribing cholestyramine to patients who may also be volume depleted, in renal failure, or taking spironolactone.

## 1. Introduction

The orally administered medication cholestyramine is a nonabsorbable anion exchange resin that serves as a bile acid sequestrant. It has been used in the treatment of hypercholesterolemia, pruritus due to biliary obstruction and elevated bile acid levels, and diarrhea due to bile acid malabsorption in the setting of ileal disease or resection [1]. Adverse effects are uncommon, though typical gastrointestinal reactions include constipation, nausea, and flatulence [1]. A handful of reports have described the rare complication of metabolic acidosis [1–11]. The following case adds to this literature in describing the occurrence of hyperchloremic nonanion gap metabolic acidosis in a 45-year-old male liver transplant patient on cholestyramine for pruritus who developed acute kidney injury.

## 2. Case Presentation

A 45-year-old Caucasian man was admitted to hospital for an elective biliary drain insertion for his recurrent bile duct stricture since a liver transplant seven years earlier. Because of the chronic cholestasis, he had been taking cholestyramine 4 g PO TID for his pruritus.

Days after the biliary drain insertion, the patient developed acute kidney injury (creatinine of 261 *μ*mol/L), in the setting of an* Enterococcus faecium* bacteremia. At this time, the serum pH was 7.09 and serum electrolytes were as follows: sodium 140 mmol/L, potassium 3.9 mmol/L, chloride 118 mmol/L, and bicarbonate 12 mmol/L (anion gap 10 mmol/L). The urine pH was 5.5 and the urinalysis was positive for bilirubin, proteinuria (1.0 g/L), and heme-granular casts. The urine electrolytes were as follows: sodium 48 mmol/L, potassium 30 mmol/L, and chloride 57 mmol/L (urine anion gap 21 mmol/L). No phosphaturia or glycosuria was noted.

Having discontinued the cholestyramine, he was given intravenous sodium bicarbonate, and his hyperchloremic metabolic acidosis resolved.

## 3. Discussion

Cholestyramine is a resin that exchanges anions once orally administered by way of its ammonium groups. It swaps chloride anions for bile acids in the lumen of the small intestine, resulting in bile acid complexes that are excreted fecally instead of being reabsorbed in the ileum [10, 12]. This exchange causes gastrointestinal secretion of bicarbonate and absorption of chloride, mediated by the duodenal brush border's apical chloride/bicarbonate antiporter [13]. The effect of this resin on chloride and bicarbonate in the gastrointestinal tract alone does not, however, lead to hyperchloremic metabolic acidosis, since the kidneys can compensate by increasing chloride excretion and bicarbonate retention. These compensatory mechanisms are impeded in states of impaired urinary acidification such as renal insufficiency and aldosterone antagonism [10, 14], which unmask cholestyramine-induced hyperchloremia and bicarbonate loss. In this report, the patient's impaired urinary acidification, as evidenced by a positive urine anion gap, was the result of renal insufficiency.

Other reported cases of cholestyramine-induced hyperchloremic metabolic acidosis have occurred in children with renal impairment [7, 8] and volume depletion (a probable cause of renal impairment) in the setting of infection [2, 9] and diarrhea ([6, 9], see Table 1). Adult cases of metabolic acidosis in the context of cholestyramine use have also been described in the setting of renal insufficiency [1, 5, 10] and spironolactone use ([1, 4, 10, 11], see Table 1).

This case and literature review highlight the complication of hyperchloremic metabolic acidosis that may occur with cholestyramine use in adult and pediatric patients. Patients taking this medication should have their electrolytes monitored, particularly in the setting a precipitating factor such as renal failure, volume depletion, and spironolactone use. If this complication occurs along with no other identified causes, then cholestyramine should be stopped, precipitants should be addressed, and sodium bicarbonate could be administered.

## Figures and Tables

**Table 1:** Summary of the literature describing cholestyramine-induced hyperchloremic metabolic acidosis.

Age	Sex (male (M), female (F))	Serum pH	Chloride (mmol/L)	Bicarbonate (mmol/L)	Precipitating factors	Case reference
1.5 days	M	7.15	125	9	Diarrhea	[6]
4 weeks	F	—	128	19	Diarrhea	[9]
5 weeks	M	6.83	130	5.4	Volume depletion, renal failure	[8]
13 weeks	M	7.28	145	15	Upper respiratory tract infection, diarrhea	[9]
6 months	M	6.88	112	—	Upper respiratory tract infection	[2]
10.5 years	F	7.18	114	9	Renal failure	[7]
45 years	M	7.09	118	12	Bacteremia, renal failure	Case presentation
45 years	M	7.12	127	8	Renal failure	[5]
51 years	F	—	115	8	Spironolactone	[10]
57 years	M	—	122	11	Diarrhea	[10]
70 years	F	7.15	128	5	Upper respiratory tract infection, renal failure, and spironolactone	[1]
70 years	F	7.34	119	14	Spironolactone	[4]
